# Factors correlating with patients’ satisfaction after undergoing cartilage repair surgery—data from the German Cartilage Registry (KnorpelRegister DGOU)

**DOI:** 10.1007/s00264-021-05274-0

**Published:** 2021-12-07

**Authors:** Svea Faber, Nick Seiferth, Peter Angele, Gunter Spahn, Matthias Buhs, Wolfgang Zinser, Philipp Niemeyer

**Affiliations:** 1 OCM | Orthopädische Chirurgie München, Steinerstrasse 6, 812306 München, Germany; 2 Klinik Für Orthopädie Und Traumatologie, Universitätsklinikum Freiburg, Freiburg im Breisgau, Germany; 3 Sporthopaedicum Berlin, Berlin, Germany; 4 Sporthopaedicum Straubing, Straubing, Germany; 5 Sporthopaedicum Regensburg, Regensburg, Germany; 6 Klinik Für Unfallchirurgie, Universitätsklinikum Regensburg, Regensburg, Germany; 7 Praxisklinik Eisenach, Eisenach, Germany; 8 Klinik Für Unfall-, Hand- und Wiederherstellungschirurgie, Universitätsklinikum Jena, Jena, Germany; 9 Norddeutsches Knorpelcentrum, COVZ Quickborn, Quickborn, Germany; 10 St. Vinzenz-Hospital, Dinslaken, Germany

**Keywords:** Cartilage, Knee, Patient satisfaction, Outcome parameter, Regeneration, Joint preservation, ACI, Bone marrow stimulation

## Abstract

Subjective patient satisfaction is the most relevant parameter for assessing the success of treatment after orthopaedic surgery. The aim of the present study was to correlate patient-reported outcome parameters (i.e., absolute KOOS, KOOS increase) and revision-free survival with patient’s satisfaction. Furthermore, the study aimed on the identification of pre-operative factors that are associated with patient’s satisfaction after the surgery.

For the present study, 6305 consecutive patients from the German Cartilage Registry (KnorpelRegister DGOU) were analyzed. Patient characteristics and outcome were correlated with patients’ satisfaction after a follow-up of three years by Spearman correlation. *P* values < 0.05 were considered statistically significant.

Mean age was 37 ± 12.5 years, 59.7% patients were male, and 40.3% female. Most patients (46.7%) were treated with an autologous chondrocyte implantation (ACI). The strongest correlation of subjective satisfaction and the subscore quality of life (*r* = 0.682; *p* < 0.001) was found, whereas the post-operative increase in KOOS from the pre-operative value showed only a moderate correlation (*r* = 0.520; *p* < 0.001). There was also a significant correlation with the absolute KOOS value (*r* = 0.678; *p* < 0.001), the subscores pain (*r* = 0.652; *p* < 0.001), quality of life (*r* = 0.682; *p* < 0.001), and sports (*r* = 0.633; *p* < 0.001), whereas symptoms (*r* = 0.504, *p* < 0.001) and activities of daily life (*r* = 0.601; *p* < 0.001) showed a weaker correlation. Pain also correlated highly significant with the patient satisfaction 24 months after surgery (*r* =  − 0.651, *p* < 0.001). The correlation between satisfaction after the 2nd and 3rd year (*r* = 0.727; *p* < 0.001) is stronger than correlation after six months and three years (*r* = 0.422, *p* < 0.001). All pre-operative parameters show a very weak correlation (*r* < 0.1).

The use of standardized measuring instruments (KOOS and Pain) is a relevant outcome parameter in science and clinical practice, whereas absolute values represent satisfaction better than the individual increase. The subscores “pain,” “quality of life,” and “sports” represent satisfaction better than the subscores “symptoms” and “activity of daily life.” Early satisfaction has only a moderate predictive value for satisfaction after 3 years, which is of great practical relevance in particular for the assessment of potential treatment failures. It is remarkable to note that a revision surgery is only very mildly associated with increased dissatisfaction. Pre-operative factors are not reliable prediction factors for post-operative patient satisfaction.

## Introduction

Most research in orthopedic surgery is measuring outcome after certain therapies by functional outcome scores such as KOOS (Knee Injury Osteoarthritis Outcome Score), IKDC (International Knee Documentation Committee), or Tegner score [1–4]. In recent years, patients’ satisfaction received increasing interest not only to broaden the understanding of the relationships between clinical results but also to reevaluate established procedures to clarify their value to the patients [5]. In cartilage repair surgery, this trend to analyze patients’ satisfaction is also visible during recent years [6–9], but reporting on satisfaction is still not broadly established—in a systematic review by Makhni et al., it could be shown that only 30% of the studies are reporting on outcome after cartilage repair by analyzing patients’ satisfaction [10]. A low satisfaction of patients is associated with increased malpractice claims [11–13], lower referral rates along with high financial losses [14], and lower reimbursement [15]. The aim of the underlying analysis was to investigate which factors correlate most with the satisfaction reported by the patients undergoing cartilage repair in a cohort of 4986 patients and if the outcome measures we are using are eligible to report on the treatments’ success to the full extent.

## Methods

Data from the German Cartilage Registry (KnorpelRegister DGOU) were used for the present analysis. The KnorpelRegister DGOU is an observational, nation-wide, and longitudinal multi-centre registry of patients assigned for surgical treatment for cartilage defects of the knee and aims to determine real-life treatment patterns and clinical outcomes. The registry was initiated by the Working Group Clinical Tissue Regeneration of the German Society for Orthopaedics and Trauma (DGOU) in 2013. Since then, the number of sites has increased to 120. The registry is conducted in accordance with the Declaration of Helsinki and registered at germanctr.de (DRKS00005617), and the current study was approved by the Ethics-Commission of the Medical Center – University of Freiburg: EK-FR 105/13_130795).

All patients aged 18 years and above that meet the following criteria are eligible to take part in the German Cartilage Registry: surgical treatment of cartilage defects of the knee, ankle or hip joint at a participating site, signed written informed consent, and possession of a personal e-mail address.

Until July 2021, 6305 patients assigned for surgical treatment for cartilage defects of the knee had been included in the registry. For the present study, data from all patients was analyzed.

Data collection is performed using a web-based RDE System “RDE-Light” which was developed by the Clinical Trials Unit (Freiburg) as an electronic data entry interface and data management system for clinical studies and other projects in clinical research. Data are collected paperless and directly on site via an Internet browser. Forms are based on HTML and PDF format. RDE-Light is available in various languages and validated according to GAMP 5. Furthermore, it fulfils all requirements of Good Clinical Practice (GCP). Established security standards like cryptographic security protocols (SSL/TLS), user authentication protocols, and authorization concepts are applied.

After the patient signs the written informed consent, the investigator is allowed to register the patient to the database. Patient- and defect-specific parameters are reported by the treating physician at the time of surgery.

The German Cartilage Registry is supported by a grant from the “Oscar-Helene-Stiftung” and the “Deutsche Arthrosehilfe e.V.”

The baseline characteristic parameters (symptom duration, age, BMI, defect stadium, size of defect, meniscus status, number of previous surgical procedures to the joint and to the cartilage defect) and outcome parameters (KOOS (Knee Injury and Osteoarthritis Outcome Score), delta KOOS, reoperations, and pain based on the numeric rating scale (NRS)) were correlated by Spearman rank correlation with patient satisfaction three years post-operatively. Chi-square test was used to analyze categorical variables; metric variables were analyzed using Kruskal–Wallis test with a post hoc Dunn-Bonferroni test. A two-proportion *z*-test was used to compare proportions. Patients’ satisfaction was measured by a 4-item scale, 1 = unsatisfied, 2 = partially satisfied, 3 = satisfied, and 4 = very satisfied, as already used in earlier studies [7, 16, 17]. *P* values < 0.005 were considered statistically significant. SPSS statistics version 26 was used to analyze the data.

## Results

### Patients’ characteristics of the analyzed cohort

Out of 6305 patients analyzed, 59.7% patients were male and 40.3% female (see Table 1). The mean age was 37 ± 12.5 years, and the mean duration of symptoms was 25.9 ± 75.8 months. Most of the defects were located at the medial femoral condyle (39.4%), followed by retropatellar defects (29.4%). The mean defect size was 357.8 ± 242.0 mm^2^. In 46.7% of the cases, the defects were treated by ACI, followed by 14.6% with bone marrow stimulating techniques (BMS). In 4.7% of the cases debridement and in 1.8% osteochondral transplantation (OATS) were used. In 50.3% of the cases, no concomitant therapy was performed; multiple concomitant surgeries (9.5%), valgisating tibia osteotomy (7.3%), and partial meniscectomy (7.9%) were the most common concomitant therapies.

**Table 1:** Baseline patients’ characteristics of 4986 patients undergoing cartilage repair on the knee joint

	Mean		Standard Deviation
Age (years)	37.32		12.48
Defect size (mm)	357.82		241.96
Symptom duration (month)	25.92		75.76
Percentage			
Gender			
Male	59.65%		
Female	40.35%		
Defect Location			
Patella		29.44%	
Trochlea		13.34%	
MFC		39.44%	
LFC		11.67%	
MTP		1.91%	
LTP		2.10%	
Other		0.66%	
Multiple		1.44%	
Type of cartilage therapy			
Drilling		0.59%	
BMS		14.61%	
OCT		1.77%	
ACI		46.74%	
ACT + Spongiosa		7.83%	
mBMS		4.06%	
Debridement		4.69%	
Other		13.06%	
Multiple		6.66%	

### Overall satisfaction

**Fig. 1:**
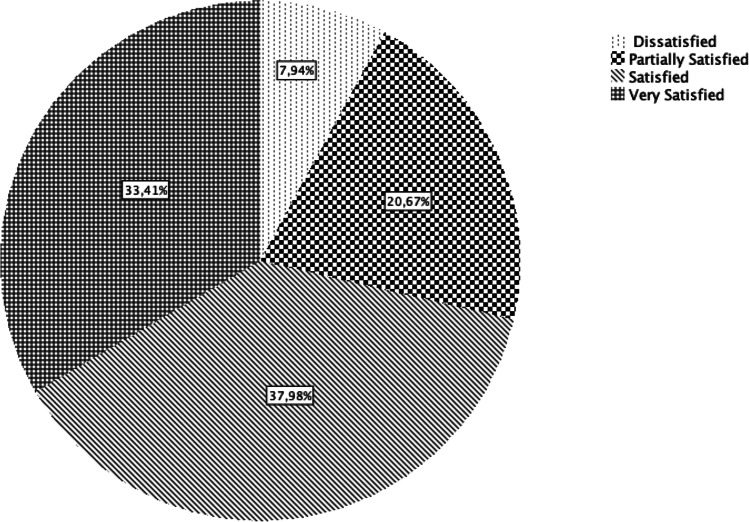
Of the patients undergoing cartilage repair, 7.9% are not satisfied, 20.7% are partially satisfied, 37.9% are satisfied, and 33.4% are very satisfied

Of the patients, 7.9% undergoing cartilage repair are not satisfied, 20.7% are partially satisfied, 37.9% are satisfied, and 33.4% are very satisfied (Fig. 1).

### Comparison of “very satisfied” and “unsatisfied” patients

Very satisfied patients are more often male (*p* = 0,003), had fewer previous operation to the cartilage (*p* = 0.007), lower pain (VAS), and higher KOOS values (all *p* < 0.001). There was no difference in symptom duration, BMI, defect size, age, or number of previous operations to the joint.

### Correlation with KOOS and re-operations at a three year follow-up

The strongest correlations of subjective satisfaction and the KOOS subscore quality of life (*r* = 0.682; *p* < 0.001), as well as with the absolute KOOS value (*r* = 0.678; *p* < 0.001), were found. An almost as high correlation was shown with the subscores pain (*r* = 0.652, *p* < 0.001) and sports (*r* = 0.633; *p* < 0.001) (Table 2). The correlation with the subscores symptoms (*r* = 0.504, *p* < 0.001) and activities of daily life (*r* = 0,601; *p* < 0.001) was a little weaker. The pain value (NRS) also correlated highly significant with the patient satisfaction (*r* =  − 0.651, *p* < 0.001). The post-operative increase in KOOS from the pre-operative value showed only a moderate correlation (*r* = 0.520; *p* < 0.001).

**Table 2:** Correlation of pre-operative values with patients’ satisfaction at the 3-year follow-up

	*r* value	*p* value
Age	− 0.056	0,017
BMI	0.027	0,253
Duration of symptoms	− 0.097	< 0.001
Number of previous surgeries to the joint	− 0.081	0.001
Number of previous surgeries to the cartilage	− 0.047	0.046

There is a highly significant negative correlation with the re-operation rate, but its correlation is very weak (*r* =  − 0.187; *p* < 0.001).

Patients undergoing bone marrow stimulation (BMS) or autologous chondrocyte implantation (ACI) have similar correlations of post-operative values with their satisfaction than the overall cohort of patient who underwent cartilage repair (Tables 3 and 4).

**Table 3:** Correlation of post-operative values with patients’ satisfaction at the 3-year follow-up

	*r* value	*p* value
KOOS total	0.678	< 0.001
KOOS ADL	0.601	< 0.001
KOOS Pain	0.652	< 0.001
KOOS QoL	0.682	< 0.001
KOOS symptoms	0.504	< 0.001
KOOS sports/rec	0.633	< 0.001
Pain	− 0.651	< 0.001
Reoperation	− 0.187	< 0.001
Satisfaction 6 M	0.422	< 0.001
Satisfaction 24 M	0.727	< 0.001

**Table 4:** Correlation of post-operative values with patients’ satisfaction at the 3-year follow-up of patients undergoing ACI

	*r* value	*p* value
KOOS total	0.693	< 0.001
KOOS ADL	0.607	< 0.001
KOOS Pain	0.656	< 0.001
KOOS QoL	0.703	< 0.001
KOOS symptoms	0.511	< 0.001
KOOS sports/rec	0.649	< 0.001
Pain	− 0.651	< 0.001
Reoperation	− 0.224	< 0.001
Satisfaction 6 M	0.445	< 0.001
Satisfaction 24 M	0.762	< 0.001

### Correlation of early with late patients’ satisfaction

There is a strong correlation between satisfaction after the 2nd and 3rd years (*r* = 0.727; *p* < 0.001), but only a moderate correlation between satisfaction after six months and after three years (*r* = 0.422, *p* < 0.001) (Table 2).

### Correlation with pre-operative baseline values

The correlation with the patients’ characteristics (age, BMI, pre-operative symptom duration, meniscus status, defect stadium, defect size, number of previous surgical procedures to the joint and to the cartilage defect) were very poor (*r* <  − 0.1) (Table 5).

**Table 5:** Correlation of post-operative values with patients’ satisfaction at the 3-year follow-up of patients undergoing ACI

	*r* value	*p* value
KOOS total	0.682	< 0.001
KOOS ADL	0.617	< 0.001
KOOS Pain	0.68	< 0.001
KOOS QoL	0.684	< 0.001
KOOS symptoms	0.511	< 0.001
KOOS sports/rec	0.618	< 0.001
Pain	-0.670	< 0.001
Reoperation	-0.057	0.314
Satisfaction 6 M	0.360	< 0.001
Satisfaction 24 M	0.702	< 0.001

## Discussion

There has been an ongoing debate on the most appropriate way to measure the outcome of orthopaedic surgery and to estimate efficiency. Since there is no reliable and strong correlation of imaging, i.e., by the means of MRI scoring systems and clinical outcome in cartilage repair procedures [18], patient-reported outcome parameters have been accepted to represent the gold standard endpoint for evaluating efficiency of cartilage repair procedures [19]. This has been underlined by a recent recommendation given by the International Cartilage Research and Joint Preservation Society (ICRS) [20]. Nevertheless, principle of these instrument is to measure activity level, pain level, and other parameters as objective as possible using standardized scoring systems. On the other hand, these scoring systems ignore important factors such as expectations of the patient. For cartilage repair patients, expectations are quite demanding but they also really vary in between individual patients [21]. Although these expectations really differ from patient’s expectations associated with other orthopaedic surgery such as total knee replacement (TKA), some patient-reported outcome measures are used such as the KOOS are used. Therefore, it seems likely that there might not be a strict correlation between absolute score values and individual satisfaction [22]. Furthermore, there is a debate if patient-reported outcome does really represent factors that are most relevant for the patients or if indivualization of these scoring systems is required [23].

The purpose of this study was to examine factors correlating with patients’ satisfaction in a large cohort of more than 6000 patients undergoing cartilage repair of the knee joint. The main findings of the underlying analysis were that only the minority of patients (7.9%) undergoing cartilage repair are dissatisfied. The usual outcome scores (KOOS and pain) represent the patients’ satisfaction the best, whereas the KOOS subscore “quality of life” has the highest correlation. Interestingly the individual increase (Delta KOOS) shows a smaller correlation and the reoperation rate the smallest. Satisfaction after 6 months has only a moderate predictive value for satisfaction after 36 months. Preoperative values (age, BMI, duration of symptoms, number of previous surgery to the joint and to the cartilage) are not eligible to predict the post-operative satisfaction of patients undergoing cartilage repair to the knee.

Health-care institutions are rating quality of medical care used for calculating reimbursement based on patients’ satisfaction [15]. A satisfied patient is more likely to recommend the doctor to others [14] and is less likely to sue the doctor for malpractice [11–13], which leads to the fact that a high post-operative satisfaction is of tremendous interest not only of the patient itself but also of the surgeon in an ethical and financial way and should be brought into focus more. Sadly only 30% of the studies analyzing outcome after cartilage repair consider satisfaction in their outcome rating [10]. In our analysis, it came up that pain, absolute KOOS value, and the KOOS subscores correlate most with the satisfaction value, meaning a pain-free and highly functional patient is a satisfied patient. Interestingly the highest correlation could be seen with the “quality of Life” subscore (Table 2), which has been shown as a factor being significantly higher in patients who underwent ACI than those who underwent BMS [24, 25]. Similar results were also reported by Tírico et al. proving lower pain levels and higher KOOS and IKDC scores in satisfied patients undergoing osteochondral allograft transplantation in the knee [26]. The question must also be asked whether patients report a higher KOOS value because their satisfaction is high, or are patients more satisfied because they have only little symptoms, a satisfying return to sport and a high quality of life? Either way it is mutually dependent, and when functional criteria included in the KOOS can be increased, the satisfaction will also increase. Interestingly the increase in KOOS correlates less with the satisfaction, than absolute KOOS values, which means that the absolute outcome is more important to the patients that the individual gain in function. In a recent analysis, similar differences especially for women could be shown; even though women have a higher gain in KOOS compared to pre-operative values, they occur to be less satisfied than men [16]. In this analysis, we could show again that the “very satisfied” patients are more often male. It is remarkable that the reoperation rate showed the lowest correlation, whereas reintervention is defined as an endpoint and/or failure in multiple studies investigating success after cartilage repair [27–33].

The appropriate measurement of patient satisfaction is ambiguous because of its multidimensional construct. Even though many instruments have been designed to measure satisfaction, they are unvalidated or rudimentary [5]. For the German Cartilage Registry, an easy-to-understand 4-item-scale was implemented (1 = unsatisfied, 2 = partially satisfied, 3 = satisfied, 4 = very satisfied) and has successfully been used already [7, 16, 17]. With this analysis, a correlation with functional outcome scores could be shown, which validates the score to some extent. Even though the vast majority of the patients was “very satisfied” or “satisfied,” we still need to consider almost 8% of dissatisfied patients three years after the operation. Interestingly, a rate of 7.9% is less compared to failure rates described by other endpoints including such as the need of revision surgery or non-responder evaluations that are based on a lack of improvement in patient-reported outcome measures [34]. Pestka described a revision rate of cartilage repair patients of 3.3% even within the first year after surgery; others described a long-term failure rate of 20% [35, 36]. Given the fact that individual expectation contributes to the rate of 8% patients that reported “unsatisfaction” in this study, these numbers are hard to compare but gives room for interpretation. Interestingly, need for revision surgery in the present study could not be associated with a lower rate of satisfaction. Furthermore, a high percentage of patients underwent previous operations to the knee and the cartilage, and the latter was significantly more often in dissatisfied patients. Maybe—compared to other studies describing outcome of primary cartilage repair studies—this might even negatively influence satisfaction rate in the present analysis. Taken altogether, it seems hard to really compare and to interpret a rate of 8% unsatisfied patients found in the present study—neither based on pre-operative nor on post-operative factors.

The goal of various studies was to predict patients’ outcome relying on pre-operative values [37–41]. The data of the underlying study reveals that the analyzed pre-operative factors represent a poor predictive value for patients’ satisfaction two years after cartilage repair. This finding was also made by the group of Baker et al. trying to predict satisfaction in patients undergoing total knee replacement by pre-operative variables [42], which means that either the pre-operative prediction is impossibly or other, here not analyzed, parameters need to be taken under consideration. A relevant factor influencing patients’ satisfaction and self-assessed rating of the post-operative outcome, which was not addressed in this analysis, is the pre-operative expectation [43–45], which is demanding and high in patients undergoing autologous chondrocyte implantation of the knee [21]. To prevent that expectation bias, surgeons should invest time in pre-operative information to help to develop realistic expectations about the impact of cartilage repair surgery. Other pre-operative values having negative influence on the post-operative satisfaction are psychological factors such as life satisfaction [46]. Furthermore, interpersonal aspects, such as physician–patient-communication, continuity of care, or waiting times, play an important but hard to measure role in subjective satisfaction [11]. As we know from earlier analysis, gender is a pre-operative factor influencing patients’ satisfaction, such that women report generally worse satisfaction rates than men [16]. Other “hard facts” such that defect size or age are easier to assess but do not predict patients’ satisfaction appropriately, which in turn also means that patients have, regardless of their patients’ and cartilage lesions’ specific pathologies, equal chances to be fully satisfied in the long term. These findings question whether preoperative thresholds may be loosened since those factors only have a little influence of post-operative satisfaction. Especially an early unsatisfied patient does not predict dissatisfaction in the long term, since satisfaction after six months has only a moderate predictive value for satisfaction after 36 months, which is of great practical relevance for the assessment of possible treatment failures. Similar results for the non-existing relation of early (6 months) and late (36 months) poor IKDC scores could be shown by Pestka et al. [3].

## Limitations

There are some significant limitations of the present study. This study analyzed patient satisfaction at a three year follow-up, which is in context of cartilage repair rather short. Another possible bias for PROMS in general is that patients gave up physical activities after the operation and therefore have higher scores and less pain. Due to the natural character of a registry input, errors on the doctor and patient side are unavoidable. Nevertheless, this is a limitation which is not specific for the present study and associated with remote data entry systems. On the other hand, remote data entry reduces bias and therefore increases study quality regarding this important point. Therefore, the major limitation of this study is the lack of a standardized and previous validated scoring system for evaluation of patient satisfaction. We decided to use a very global rating scale just discriminating between four different grades of patient’s satisfaction ranging from “very satisfied” to “unsatisfied.” Correlation of this rating system was analyzed with KOOS and NRS pain score. This correlation at least represents a partial validation of this rating scale for the subgroup of patients who underwent cartilage repair. Nevertheless, since correlations to important subscore were found in the present study and since the rating scale used in the present study proofed to be efficient in the German Cartilage Repair, a systematic validation study of this rating scale should be initiated in the future.

## Conclusion

The use of standardized measuring instruments (such as the KOOS) is a relevant outcome parameter in science and clinical practice, whereas absolute values represent satisfaction better than the individual increase. The subscore “quality of life” correlates the best with patients’ satisfaction, also “pain” and “sport” represent satisfaction better than the subscores “symptoms” and “activity of daily life” in the collective of patients after cartilage regenerative interventions. Early satisfaction has only a moderate predictive value for satisfaction after 36 months, which is of great practical relevance in particular for the assessment of possible treatment failures. It is remarkable to note that a revision surgery is only very mildly associated with increased dissatisfaction. Pre-operative values such as age, BMI, number of previous operations, and duration of symptoms before the operation show no correlation with patient satisfaction after cartilage regenerative interventions.
